# Ub and Dub of RNF43/ZNRF3 in the WNT signalling pathway

**DOI:** 10.15252/embr.202152970

**Published:** 2021-05-03

**Authors:** Gabriele Colozza, Bon‐Kyoung Koo

**Affiliations:** ^1^ Institute of Molecular Biotechnology of the Austrian Academy of Sciences (IMBA) Vienna Biocenter (VBC) Vienna Austria

## Abstract

The E3 ubiquitin ligases RING finger protein 43 (RNF43) and zinc and RING finger 3 (ZNRF3) have received great attention for their critical role in regulating WNT signalling during adult stem cell homeostasis. By promoting the turnover of WNT receptors, Frizzled and LRP5/6, RNF43 and ZNRF3 ensure that proper levels of WNT activity are maintained in stem cells. The molecular mechanism of RNF43/ZNRF3 activity is beginning to emerge from several recent studies, yet little is known about the regulation of RNF43/ZNRF3 at the post‐translational level. A study in this issue of *EMBO Reports* identifies the deubiquitinating enzyme USP42 as a key regulator of WNT signalling, which acts by antagonizing the ubiquitin‐dependent clearance of RNF43/ZNRF3 induced by R‐spondins (Giebel *et al*, 2021).

## Graphical abstract

A study in this issue implicates the deubiquitinating enzyme USP42 in Wnt signalling. USP42 stabilizes RNF43/ZNRF3 at the cell surface, resulting in increased turnover of LRP6 and Frizzled and the inhibition of Wnt signalling.

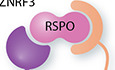

WNT/β‐catenin signalling (also known as canonical WNT signalling) is an evolutionarily conserved pathway involved in embryonic development of multicellular animals (Nusse & Clevers, 2017). In adult tissues, canonical WNT signalling controls self‐renewal and maintenance of a variety of tissue‐specific stem cells, most notably the intestinal stem cells (ISCs) residing at the bottom of the intestinal crypt (Nusse & Clevers, 2017). In order to maintain a correct balance between stem cell proliferation and differentiation, as in typical homeostatic conditions, WNT/β‐catenin signalling needs to be tightly regulated. Several mechanisms have evolved to prevent WNT overactivation. Genetic mutations that override these safe‐guard mechanisms, leading to sustained WNT activation, are often at the basis of different types of cancers. An example is colorectal adenocarcinoma, which is most frequently initiated by loss‐of‐function mutations in the WNT negative regulator, adenomatous polyposis coli (APC).

The two related paralogues RNF43/ZNRF3 (hereafter R/Z) represent a class of single‐pass transmembrane RING‐type E3 ubiquitin ligases that attenuate WNT signalling at the plasma membrane, by promoting ubiquitination and endo‐lysosomal degradation of FZD and LRP5/6 (Hao *et al,* 2012; Koo *et al,* 2012). Both proteins contain a signal peptide, an extracellular protease‐associated (PA) domain, a transmembrane domain and an intracellular RING domain with catalytic activity. R/Z are expressed in different types of stem cells, including ISCs, and are induced by WNT, forming a negative feedback loop that prevents uncontrolled stem cell proliferation. R/Z function is of fundamental importance for keeping stem cells in check, as evidenced by double knockout mice which show strong over‐proliferation of the intestinal stem cells and formation of tumours, similar to APC mutations (Koo *et al,* 2012). R/Z mutations are indeed found in various human cancers, including colorectal and pancreatic carcinomas. Differently from APC, however, R/Z tumours still depend on a source of WNT ligands and are sensitive to PORCN inhibitors, small molecules that prevent WNT secretion and inhibit paracrine signalling (Koo *et al,* 2015). R/Z activity is also finely regulated, particularly by leucine‐rich repeat‐containing G protein‐coupled receptor (LGR) 4/5/6, which binds secreted WNT agonists R‐spondins (Rspo) and forms a ternary complex with R/Z, promoting their membrane clearance through an auto‐ubiquitination mechanism (de Lau *et al,* 2011; Hao *et al,* 2012). So, by removing R/Z, Rspo increases FZD and LRP5/6 at the cell surface, boosting WNT signalling (Fig 1). Other players have been shown to modulate R/Z activity. Surprisingly, the cytosolic scaffold protein dishevelled (DVL), an essential component of the WNT pathway, is also required for R/Z activity, by recruiting the E3 ligases to WNT receptors and promoting their ubiquitination‐dependent degradation (Jiang *et al,* 2015). More recently, several key studies showed that R/Z activity is regulated by post‐translational modifications. For example, casein kinase 1 (CK1)‐dependent phosphorylations were shown to be crucial for regulating RNF43 function (Spit *et al,* 2020; Tsukiyama *et al,* 2020), while the phosphatase PTPRK was shown to promote ZNRF3 activity by keeping it unphosphorylated (Chang *et al,* 2020).

**Figure 1:**
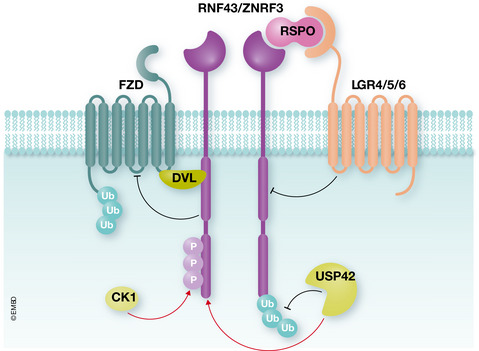
The deubiquitinating enzyme USP42 stabilizes RNF43/ZNRF3 at the plasma membrane When bound to RSPO and LGR, RNF43/ZNRF3 are removed from the cell surface as a result of auto‐ubiquitination and endocytosis, inhibiting the E3 ligase activity. USP42 counteracts R/Z auto‐ubiquitination, stabilizing their levels on the plasma membrane. Consequently, R/Z are available to inhibit WNT signalling by promoting FZD ubiquitination and endo‐lysosomal degradation. CK1 phosphorylates R/Z cytoplasmic tail, enhancing its anti‐WNT activity. DVL bridges R/Z to FZD, allowing downregulation of the WNT receptors.

The present work from Giebel *et al* (2021) adds another fundamental piece to the complex puzzle of R/Z regulation. The authors first performed a siRNA screening against deubiquitinating enzymes (DUBs) in HEK293T cells, using the well‐established TOPflash luciferase reporter as a readout for WNT/β‐catenin signalling. The authors identified ubiquitin‐specific protease 42 (USP42) as a negative regulator of WNT signalling, since its knock‐down upregulated WNT‐induced luciferase activity. Interestingly, USP42 knock‐down cooperated with Rspo treatment in the WNT reporter assay but, critically, did not further increase WNT signalling in R/Z double KO cells, suggesting that R/Z are epistatic to USP42. Further biochemical analysis revealed that USP42 knock‐down increased, while its overexpression diminished, R/Z polyubiquitination. Importantly, a catalytically inactive mutant USP42^C120A^ was unable to inhibit WNT signalling, validating the idea that USP42 functions in the WNT pathway through de‐ubiquitination of R/Z (Fig 1). Interaction between USP42 and R/Z was confirmed by co‐immunoprecipitation, and using different deletion constructs, the authors could map the binding in the Dvl interacting region (DIR), found in the cytoplasmic domain of R/Z and already known to play an important regulatory role (Jiang *et al,* 2015). On the other hand, USP42 required an intact N‐terminal domain and catalytic activity for its interaction with ZNRF3, while other regions were dispensable. Elegant cell‐surface biotinylation experiments showed that USP42 overexpression reduced the ubiquitin “earmarks” on R/Z, while increasing R/Z plasma membrane levels. Notably, co‐expression of R/Z and USP42 decreased cooperatively FZD levels, through an active endocytic process, while knock‐down of USP42 increased endogenous LRP6 levels in cultured cells. Altogether, the evidence brought by Giebel and colleagues shows that USP42 synergizes with R/Z in promoting WNT receptor turnover. Perhaps the most significant insight at a mechanistic level was that USP42 stabilized the formation of a ternary complex comprising ZNRF3, Rspo and LGR4, preventing at the same time ubiquitin‐dependent clearance of ZNRF3 induced by Rspo/LGR. This led the authors to suggest that USP42 directly oppose Rspo/LGR‐mediated clearance of R/Z, by keeping the E3 ligases in a de‐ubiquitinated form and stalling the ternary complex between Rspo, LGR and R/Z.

USP42 mRNA is often upregulated in various cancers. To understand its role in cancer cells, Giebel *et al* (2021) performed bulk‐RNA sequencing on HCT116 colorectal cancer cells upon USP42 knock‐down, showing upregulation of genes associated with intestinal stem cell features as well as epithelial–mesenchymal transition (EMT). The latter was corroborated by loss of cell–cell adhesion molecule E‐cadherin, whose expression could be rescued by treatments that inhibited WNT secretion. Thus, loss of USP42 confers cancer cell EMT characteristics and hypersensitivity to WNT signalling, a finding paralleled by experiments on mouse intestinal organoids. Wild‐type organoids died when Rspo was withdrawn from culture medium; however, USP42 KO intestinal organoids could still grow, indicating independency from Rspo factors, as a consequence of hypersensitivity to WNT. However, when endogenous WNT secretion was blocked, USP42 KO organoids also died, confirming their growth advantage still depended on a source of paracrine WNT signalling, similar to R/Z double KO organoids (Koo *et al,* 2012; Koo *et al,* 2015). Altogether, the work from Giebel and colleagues defines a novel role for USP42 in WNT signalling and reveals important insights into its molecular function as a key factor that stabilizes R/Z at the cell surface. However, some key questions remain still open. For example, the precise role of USP42 in the intestinal stem cells or other tissue‐specific stem cells was not investigated. Due to the importance of R/Z and WNT in stem cell regulation, it is tempting to speculate that USP42 (or perhaps other cell type‐specific DUBs) may play a fundamental role in stem cell homeostasis. Another emerging point is the importance of the cytosolic tail of R/Z in modulating the E3 ligase activity, by integrating different types of post‐translational modifications. Elucidating how different modifications are coordinated on R/Z will provide fundamental insights on the complexity of its regulation.
